# Identifying Aberrant 1CM-Related Pathways by Multi-Omics Analysis and Validating Tumor Inhibitory Effect of One-Carbon Donor Betaine in Gastric Cancer

**DOI:** 10.3390/ijms26083841

**Published:** 2025-04-18

**Authors:** Jie Li, Huan Liu, Panpan Yang, Feng Zhu, Fei Shen, Geyu Liang

**Affiliations:** 1 Key Laboratory of Environmental Medicine Engineering, Ministry of Education, School of Public Health, Southeast University, Nanjing 210009, China; lijies7@163.com (J.L.); huanliu2001@163.com (H.L.); yangpanpan199912@163.com (P.Y.); 2 Jiangsu Provincial Center for Disease Control and Prevention, 172 Jiangsu Rd, Nanjing 210009, China; zf850824@163.com (F.Z.); njutcmsf@126.com (F.S.)

**Keywords:** gastric cancer, multi-omics, one-carbon metabolism, betaine, transcriptomics

## Abstract

Metabolic reprogramming, a well-established hallmark of gastric carcinogenesis, has been implicated in driving tumor progression. Nevertheless, the precise mechanisms through which these metabolic alterations orchestrate gastric cancer (GC) pathogenesis remain incompletely elucidated. We conducted metabolomic analyses of plasma samples obtained from 334 patients with GC and healthy individuals to identify differential metabolites and metabolic pathways. Transcriptome sequencing was conducted on six pairs of tissues, and a joint analysis of the transcriptome and metabolome was performed. Single-cell sequencing data were acquired and co-analyzed with metabolomics to investigate metabolic abnormalities at the single-cell level. Finally, four representative metabolites selected using Random Forest analysis were subjected to cellular experiments to elucidate the mechanisms through which these metabolites exert their effects. Metabolomic analyses revealed that serine and glycine metabolism, glycolysis, and glutamate metabolism were significantly altered in GC, suggesting that one-carbon metabolism (1CM)-related pathways are aberrantly activated. A combined analysis of the transcriptome, single-cell transcriptome, and metabolomics indicated that pathways related to oxidative phosphorylation, nucleotide metabolism, and amino acid metabolism in epithelial cells were altered in GC. Cellular experiments demonstrated that the one-carbon donor metabolite betaine could inhibit the activity, invasion, and migration of GC cells while activating the phosphorylation of AMPKα. In conclusion, the 1CM-related pathway and the metabolite betaine play significant roles in GC, and the mechanisms through which the one-carbon donor betaine influences GC warrant further investigation.

## 1. Introduction

As a leading cause of gastrointestinal cancer mortality, gastric carcinoma imposes substantial clinical burdens, with 5-year survival rates below 30% for advanced-stage disease [1]. According to the 2022 global cancer statistics, the incidence and mortality rates of GC in China are projected to be 388,700 and 260,400, respectively, which renders the efficient prevention and treatment of GC a critical public health challenge [2]. The spatiotemporal complexity of the gastric cancer microenvironment drives profound therapeutic resistance, resulting in a stagnant 5-year survival rate below 30% despite multimodal treatment advances [3]. Therefore, a more comprehensive understanding of the underlying pathogenesis is crucial for enhancing the prognosis of GC.

Given the heterogeneity of the gastric cancer (GC) microenvironment, multi-omics technologies—ranging from transcriptomics to metabolomics—can comprehensively elucidate the pathogenesis across multiple levels [4]. Metabolic reprogramming represents one of the fundamental features of GC, with notable differences observed between the metabolic pathways in tumors and those in normal tissues [5]. Studies have demonstrated that metabolic reprogramming is closely linked to tumor development; therefore, it is essential to explore metabolism-related pathways and alterations in metabolites in greater depth. Given the complexity of tumor pathogenesis, it is essential to integrate transcriptomic and single-cell transcriptomic analyses to further investigate the relationship between gene expression and metabolic reprogramming at the single-cell level [6]. Numerous critical metabolic pathways are altered in tumors. Tumor cells exhibit significantly elevated levels of glycolysis compared to normal cells; moreover, due to the relatively inefficient energy production of glycolysis, tumor cells consume more glucose [7]. Simultaneously, tumor cells enhance amino acid uptake; additionally, the free amino acid glutamine—an essential energy source for tumor cells—can be converted to supplement the tricarboxylic acid (TCA) cycle [8,9]. Notably, in tumor cells, the consumption of glucose and glutamine alone is insufficient to sustain cell proliferation. While glutamine dependence is well-documented, non-canonical amino acids collectively supply >65% of total intracellular carbon/nitrogen flux in cancer cells, with serine particularly fueling tumorigenesis through dual roles: as a one-carbon metabolic substrate and NADPH regeneration cofactor [9,10]. Serine can be generated through the conversion of glucose via the glycolytic pathway and subsequently loses an additional carbon unit to become glycine, thus serving as a significant carbon donor for one-carbon metabolism (1CM) [11,12,13,14]. The metabolic processes associated with the production and transfer of one-carbon units are collectively referred to as 1CM. One-carbon units encompass methyl, methylene, and formyl groups, which are derived from glycine, histidine, serine, and tryptophan, with serine serving as the primary source [15]. 1CM encompasses the folate cycle, the methionine cycle, and the transsulfuration pathway, all of which facilitate the synthesis of purines, thymidine, and glutathione [16]. These intermediary metabolites regulate tumor metabolism by acting as precursors for the synthesis of proteins, lipids, and nucleotides [17]. Therefore, an in-depth study of 1CM in GC through multi-omics approaches is essential.

An additional significant role of metabolomics is the identification of differential metabolites. These metabolites serve two primary functions within the tumor microenvironment. On one hand, they supply essential nutrients necessary for the proliferation of cancer cells. Conversely, certain oncogenic metabolites may exert tumorigenic effects by altering tumor-related signaling pathways [18]. In our study, we observed a significant reduction in the metabolite betaine in the plasma of GC patients, which inhibited the viability, invasion, and migration of GC cells, as demonstrated through the ultra-high-performance liquid chromatography–high-resolution mass spectrometry (UHPLC-HRMS) technique in conjunction with molecular experiments. Betaine exhibits antioxidant properties and can inhibit the aberrant metabolic activities of tumor cells by mitigating oxidative stress [19]. As a donor of three methyl groups, betaine can also engage in 1CM via the methionine pathway [20]. Research has demonstrated that the one-carbon donor betaine enhances the SAM/SAH ratio and elevates the expression of *YTHDF2*, thereby diminishing the stability of the inflammatory vesicle *NLRP3* mRNA [21]. Additionally, betaine can mitigate energy perturbations by reducing lipid accumulation [22], optimizing glucose metabolism [23], and engaging in amino acid metabolism [24]. Research indicates that betaine may influence the energy levels of tumor cells by activating the *AMPK* signaling pathway, thereby inhibiting the aberrant metabolic activities of these cells [25]. Therefore, exploring the biological functions of differential metabolites is essential for the personalized treatment of GC.

Consequently, in this study, we employed metabolomics to identify the aberrant differential metabolic pathways and metabolites through the analysis of plasma samples. The integrated analysis of transcriptomic and single-cell transcriptomic data alongside metabolomics reveals that 1CM pathways are aberrantly expressed in GC. Cellular experiments demonstrated that the one-carbon donor betaine can inhibit the progression of GC by promoting the phosphorylation of *AMPK*. These findings underscore the relationship among metabolites, gene expression, and protein levels.

## 2. Results

### 2.1. Plasma Metabolomics Reveals Differential Metabolites Between Gastric Cancer (GC) Patients and Healthy Individuals

**Figure 1:**
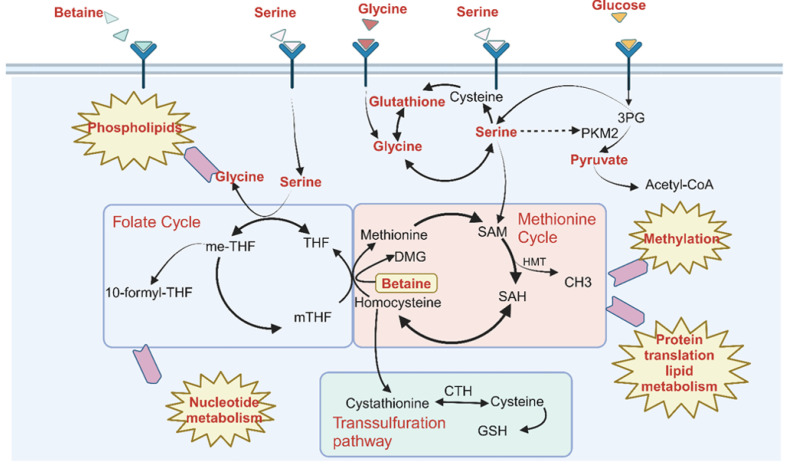
One-carbon metabolism (1CM)-related pathways are involved in various biological processes. Serine obtained through glycolysis or in vitro uptake can participate in the 1CM cycle via the methionine and folate cycles, thereby influencing pathways related to methyl metabolism, nucleotide metabolism, and lipid synthesis [25]. Glycine is involved in 1CM through its interconversion with serine [26,27,28,29,30].

**Figure 2:**
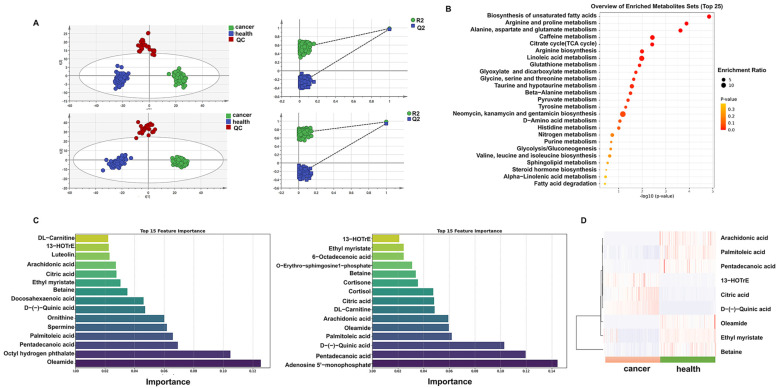
Plasma untargeted metabolomics analysis results are presented as follows: (**A**) OPLS-DA plot showing positive and negative differential metabolites; (**B**) pathway enrichment analysis plot of annotated differential metabolites; (**C**) top 15 early biomarkers and all biomarkers identified using the Random Forest method, respectively; (**D**) heatmap illustrating the 9 overlapping differential metabolites.

To reveal alterations in metabolites and metabolic pathways in the plasma of GC patients (Figure 1), an untargeted assay was conducted using the Orbitrap Exploris, and the data were processed and annotated using Compound Discoverer 3.1. PCA modeling was performed to assess the differences between GC patients and healthy individuals, with results indicating significant differences in metabolites (Figures S1 and S2). Additionally, the OrthogonalPartialLeast Squares-DiscriminantAnalysis (OPLS-DA) model demonstrated significant differences in both positive and negative modes (Figure 2A). Furthermore, 200 permutation tests confirmed that the model did not exhibit overfitting (Q^2^ < 0). According to the criteria (*p* < 0.05, |Log2FC| > 1), a total of 159 annotated differential metabolites were identified. Pathway enrichment analysis revealed significant alterations in pathways, including glycolysis, nitrogen metabolism, glutamate metabolism, purine metabolism, glutathione metabolism, and glycine-serine metabolism in GC. The glycolytic pathway contributes to one-carbon metabolism (1CM) by producing pyruvate and serine (Figure 1). One-carbon units in tumors are primarily derived from serine, and tumor cells enhance the supply of one-carbon units through serine uptake. Serine is converted to tetrahydrofolic acid, which is subsequently utilized to produce glycine and 5,10-methylenetetrahydrofolate (me-THF), providing the cells with one-carbon units (Figure 1). One-carbon metabolism-related pathways are also implicated in tumor progression by influencing purine, glutathione, nitrogen, and various amino acid metabolism (Figure 1). In conclusion, the 1CM-related pathway is significantly altered in GC compared to that of healthy individuals (Figure 2B).

To further investigate biomarkers for early GC, we analyzed plasma metabolites in stage I GC and healthy individuals. The results revealed significant differences between stage I GC and healthy controls (Figure S1). Subsequently, we statistically analyzed the early and all differential metabolites using the Random Forest method and identified the top 15 differential metabolites along with their VIP values (Figure 2C). Among these fifteen metabolites, nine were found to be significantly different in the early stages and throughout the progression of GC compared to healthy controls, including arachidonic acid, palmitoleic acid, pentadecanoic acid, 13(S)-HOTrE, citric acid, quinic acid, oleamide, ethyl myristate, and betaine (Figure 2D). These findings suggest that these nine plasma metabolites may play a crucial role in the development of GC.

### 2.2. One-Carbon Donor Metabolite Betaine Inhibits the Development and Progression of Gastric Cancer (GC) Cells

**Figure 3:**
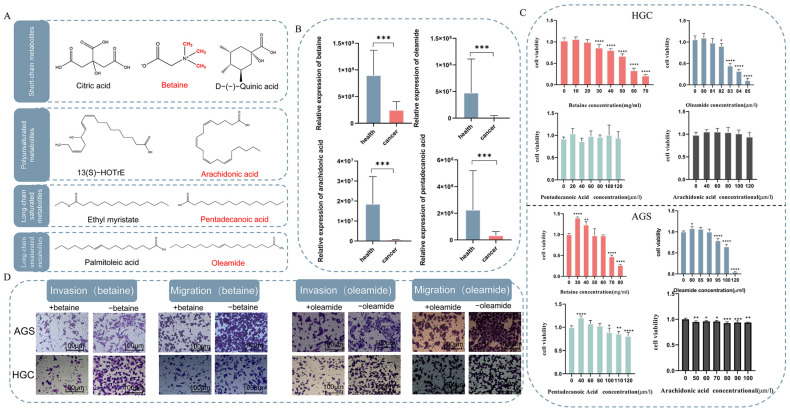
Cellular assays of differential metabolites are presented as follows: (**A**) the nine metabolites categorized into four main groups: short-chain, polyunsaturated, long-chain saturated, and long-chain unsaturated metabolites; (**B**) expression levels of four representative differential metabolites in the plasma of GC patients and the healthy pop2ulation; (**C**) results of CCK-8 experiments involving the four differential metabolites; (**D**) results of Transwell experiments involving betaine and oleamide.* *p* < 0.05, ** *p* < 0.01, *** *p* < 0.001, **** *p* < 0.0001, compared to concentration 0.

To explore the roles of differential metabolites in gastric cancer (GC), functional assays were conducted. Based on the chemical properties of the metabolites, the nine metabolites were categorized into four groups: short-chain, polyunsaturated, long-chain saturated, and long-chain unsaturated. To further investigate the biological roles of these metabolites, we selected one metabolite from each of the four categories for subsequent studies (Figure 3A), specifically betaine, arachidonic acid, pentadecanoic acid, and oleamide. Further comparisons between GC patients and healthy individuals revealed that the levels of betaine, arachidonic acid, pentadecanoic acid, and oleamide were reduced in the plasma of GC patients compared to those of healthy individuals (Figure 3B), suggesting that these metabolites may exert an inhibitory effect on GC.

The roles of four differential metabolites in gastric cancer (GC) were investigated through cell viability assays. CCK-8 experiments were conducted using both HGC-27 and AGS cells (Figure 3C). The results indicated that as the concentration of metabolites in the cell culture medium increased, an inhibitory effect on HGC-27 cells was observed (Figure 3C), with betaine demonstrating a significant inhibitory trend at a concentration of 30 mg/mL. Similarly, oleamide exhibited inhibitory effects on HGC-27 cells, with a significant effect emerging at a concentration of 83 µM. However, pentadecanoic acid and arachidonic acid did not exhibit significant inhibitory effects on HGC-27 cells, even at concentrations up to 120 µM. In AGS cells, both betaine and oleamide demonstrated similar inhibitory effects on gastric cancer cell viability with increasing concentrations, exhibiting significant inhibitory effects at 70 mg/mL and 95 µM, respectively. Unlike HGC-27 cells, pentadecanoic acid and arachidonic acid exhibited significant inhibitory effects on AGS cells, at 100 µM for pentadecanoic acid and 50 µM for arachidonic acid. In conclusion, as both betaine and oleamide can inhibit the activity of AGS and HGC-27 cells, we will further investigate the effects of these metabolites on the invasive and migratory abilities of GC cells.

The effects of betaine and oleamide on invasion and migration were assessed using a Transwell assay (Figure 3D). Different concentrations of betaine and oleamide, which did not significantly affect the viability of GC cells, were added to HGC-27 and AGS cells. The results demonstrated that both betaine and oleamide inhibited the invasion and migration of GC cells. Given that previous literature suggests that betaine, as a one-carbon donor, exerts a tumor-suppressive effect, we will next focus on the mechanism by which betaine inhibits GC.

### 2.3. Tissue Transcriptome and Single-Cell RNA Sequencing, Combined with Metabolomics, Reveal Changes in 1CM-Related Pathways in GC

**Figure 4:**
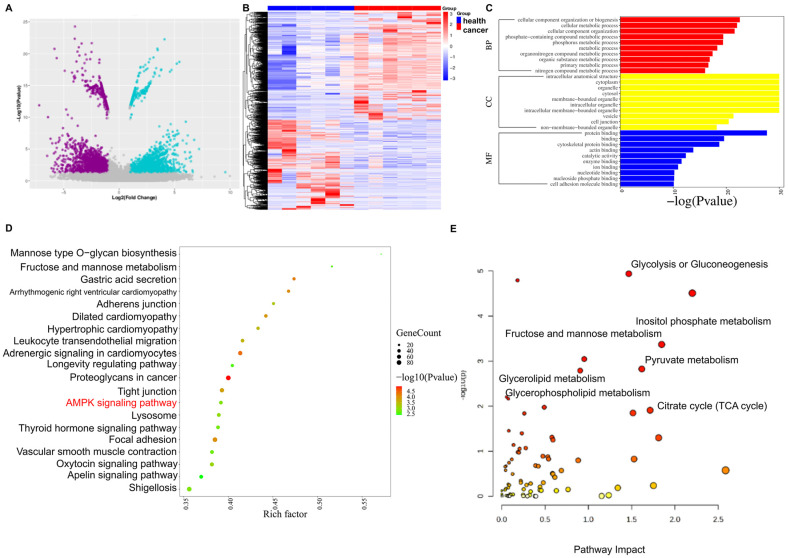
Transcriptome sequencing analysis of gastric cancer (GC) and paracancerous tissues is presented as follows: (**A**,**B**) volcano and heat maps of transcriptome sequencing results; (**C**,**D**) functional and KEGG pathway enrichment analysis of the transcriptome; (**E**) joint analysis of transcriptome sequencing and metabolomics results.

A joint analysis of transcriptome and metabolomics was conducted to investigate significantly altered signaling pathways in GC tissues. To investigate the pathogenesis of GC at the genetic level, we performed transcriptome analysis on six pairs of cancerous and paracancerous tissues obtained from GC patients. Volcano and heat maps illustrated significant differences in gene expression between GC tissues and paracancerous tissues (Figure 4A,B). Functional and Kyoto Encyclopedia of Genes and Genomes (KEGG) analyses showed that the differential genes were primarily involved in lipid and glucose metabolic processes (Figure 4C,D). Furthermore, we conducted a combined analysis of differential genes and metabolites and found that signaling pathways associated with one-carbon metabolism (1CM), including glycolysis, glucuronogenesis, the phosphatidylinositol signaling system, pyruvate metabolism, and TCA cycling, were significantly altered in GC tissues (Figure 4E).

**Figure 5:**
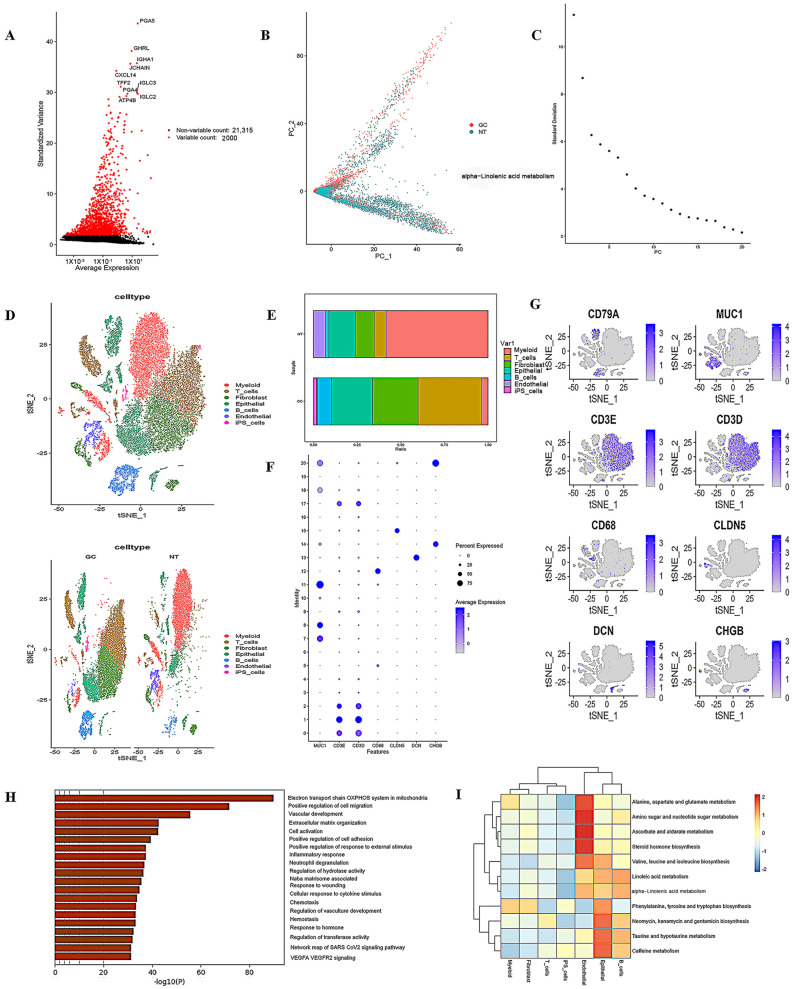
Results of the combined single-cell sequencing and metabolomics analysis of gastric cancer (GC) are presented as follows: (**A**) volcano plots of highly variable genes; (**B**,**C**) principal component analysis (PCA) plots for single-cell sequencing; (**D**) t-SNE plots for cell type identification; (**E**) bar plots illustrating the proportion of cell types in each sample; (**F**) dot plot of cell type-specific marker genes across all cell types; (**G**) expression levels of *CD79A*, *MUC1*, *CD3E*, *CD3D*, *CD68*, *CLDN5*, *DCN*, and *CHGB* mRNAs; (**H**) pathway enrichment analysis of single-cell sequencing; (**I**) pathway enrichment analysis based on combined single-cell sequencing and metabolomics.

To further understand the changes in gastric carcinogenesis at the single-cell level, we retrieved single-cell transcriptome data for GC from the GEO database (https://www.ncbi.nlm.nih.gov/ accessed on 15 July 2024) for analysis. All single-cell RNA sequencing (scRNA-seq) data were analyzed following integration using the Seurat software package (Seura.v5). The results indicated that most cells had nFeature_RNA values below 5000, nCount_RNA values below 20,000, and percent.mt values below 20% (Figure S3). We conducted data quality control using the criteria of 200 < nFeature_RNA < 2500 and percent.mt < 20%. Filtered cells were subsequently analyzed, and highly variable genes were selected based on the variance in gene expression. The 2000 genes with the highest variance were selected for further analysis (Figure 5A). We then applied PCA to reduce the dimensionality of the data and generate PCA plots (Figure 5B). Additionally, we utilized the Elbow Plot to rank the principal components according to their standard deviation (Figure 5C). This analysis indicated that principal components 1 to 12 (PC1 to PC12) effectively represented the highly variable genes (Figure S4). Next, seven cell types (myeloid, T cells, fibroblasts, epithelial, B cells, endothelial, and iPS cells) were identified using the t-SNE algorithm to nonlinearly reduce the dimensionality of the principal components based on the annotation of classical marker genes (Figure 5D). We found that GC tissues exhibited a clear decrease in endothelial cells and an increase in epithelial and T cells (Figure 5D,E). The expression of marker genes for each cell type and their distribution across different cell types are shown (Figure 5F,G).

Pathway enrichment analysis revealed that the oxidative phosphorylation pathway in mitochondria was the most significantly altered pathway in gastric cancer (GC) (Figure 5H). Next, we jointly analyzed the transcriptome results alongside metabolomics at the single-cell level and found that the synthesis of amino acids (Figure 5I: biosynthesis of valine, leucine, isoleucine, phenylalanine, tyrosine, and tryptophan) was significantly increased in epithelial cells in GC compared to normal tissues. These amino acids are essential and can participate in the one-carbon metabolism (1CM) process of tumors through the production of acetyl coenzyme A, succinic acid, and tetrahydrofolate. In conclusion, the combined analysis of single-cell sequencing and metabolomics revealed that the oxidative phosphorylation and 1CM pathways in mitochondria were significantly altered in GC tissues.

### 2.4. One-Carbon Donor Betaine Could Inhibit GC by Activating AMPK Phosphorylation

**Figure 6:**
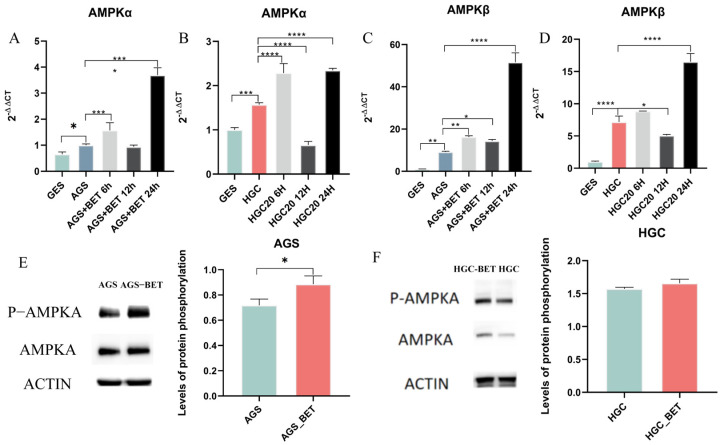
(**A**–**D**) mRNA expression levels of *AMPKα* and *AMPKβ*; (**E**,**F**) expression levels of *AMPKα* and its phosphorylated proteins. * *p* < 0.05, ** *p* < 0.01, *** *p* < 0.001, **** *p* < 0.0001.

Existing studies have demonstrated that the one-carbon donor betaine can activate AMP-activated protein kinase (*AMPK*) by increasing methionine concentrations. *AMPK*, a member of the serine/threonine kinase family, influences the supply of one-carbon units by regulating enzymes associated with tetrahydrofolate (THF) metabolism, such as tetrahydrofolate reductase. The transcriptome sequencing results of the present study also indicated that the *AMPK* signaling pathway was aberrantly perturbed in gastric cancer (GC). Therefore, we will investigate whether the addition of betaine, a one-carbon donor in the one-carbon metabolism (1CM) pathway, inhibits gastric cancer cells by influencing the phosphorylation of the *AMPK* signaling pathway. Betaine was added to HGC-27 (20 mg/mL) and AGS (50 mg/mL) cells, respectively, and qPCR experiments revealed that the mRNA levels of *AMPKα* and *AMPKβ* were significantly elevated after 24 h of betaine incubation (Figure 6A–D). Further West ern blot (WB) experiments demonstrated that the addition of betaine increased the phosphorylation levels of *AMPKα* compared to those in cells without betaine (Figure 6E,F). These results suggest that betaine may inhibit the progression of GC by enhancing the phosphorylation levels of *AMPK* protein.

## 3. Discussion

Metabolic reprogramming is a crucial marker in tumor progression and metastasis [31]. The metabolism of cancer cells is reprogrammed to meet the energy and nutrient demands for rapid proliferation and metastasis [32]. Due to the heterogeneity of gastric cancer (GC) and the complexity of its pathogenesis, the use of multi-omics technology aids in exploring the pathogenesis of GC at multiple levels, from genes to metabolites. Pathway enrichment analysis using metabolomics revealed that amino acid metabolism and energy metabolism pathways, closely related to 1CM, were altered in GC [33]. The joint analysis of transcriptome and metabolome data can explore the intrinsic changes of organisms from the levels of “cause” and “effect”, targeting key pathways of metabolite changes and elucidating biological problems. In this study, the combined analysis of these two sequencing results demonstrated that 1CM-related pathways, including glycolysis, TCA cycle, and pyruvate metabolism, were significantly altered in GC. Additionally, pathway enrichment analysis of the transcriptome revealed that the energy-regulation-related AMP-activated protein kinase (*AMPK*) signaling pathway was also significantly altered in GC tissues. This finding is consistent with previous studies that demonstrated significant alterations in energy metabolism pathways, such as glycolysis, in GC [34]. Further single-cell transcriptome and metabolome analyses indicated that 1CM pathways, such as amino acid metabolism, were significantly altered in GC endothelial and epithelial cells, with the oxidative phosphorylation metabolism pathway being the most significantly altered pathway in GC. This observation aligns with the findings of Sun et al. (2023), which demonstrated that the metabolic pathways of oxidative phosphorylation, amino acid synthesis, and lipid synthesis were significantly altered in GC [35]. In conclusion, combined transcriptomic and metabolomic analyses revealed that 1CM-related pathways were significantly altered in GC.

Betaine plays a crucial role in one-carbon metabolism (1CM) as a one-carbon donor that contains three methyl groups. Previous studies on the metabolomics of gastric cancer (GC) have demonstrated significant alterations in metabolites of GC compared to healthy individuals. However, few studies have reported significant alterations in plasma betaine concentrations among patients diagnosed with gastric cancer. Moreover, recent studies have found that there is an important correlation between nutrition and tumor prognosis, and the addition of betaine as an additive food agent is therefore of great significance in the prevention and treatment of tumors [36]. In 2018, Zhang et al. published an article indicating that betaine levels were significantly reduced in gastric cancer patients with lymph node metastasis [37], suggesting its potential role as a diagnostic and prognostic factor. In this study, we found that betaine is expressed at notably low levels in the plasma of gastric cancer patients; furthermore, the random forest approach identified it as one of the top 15 potential biomarkers. Previous studies have demonstrated that betaine plays a significant role in pathways associated with one-carbon metabolism (1CM). Betaine may reduce lipid accumulation by upregulating betaine-homocysteine methyltransferase (BHMT) expression and influences cellular energy through an increase in the SAM/SAH ratio [21]. Additionally, betaine acts as a one-carbon donor, exerting an inhibitory effect on inflammation by influencing the synthesis of amino acids, including methionine [38]. It is also involved in amino acid synthesis associated with 1CM by directly influencing homocysteine concentrations [39,40]. Moreover, the addition of betaine reduces lipid levels via *AMPK* activation [25]. However, there is a paucity of studies investigating the mechanisms underlying the inhibitory effects of betaine in gastric cancer. Our experimental results indicate, for the first time, that betaine inhibits the activity, invasion, and migration of gastric cancer cells while promoting *AMPKα* protein phosphorylation. It has been demonstrated that the *AMPK* signaling pathway influences amino acid and lipid metabolic pathways, playing a pivotal role in regulating cellular energy homeostasis by enhancing the efficiency of oxidative phosphorylation [41,42,43]. Thus, our findings suggest that the activation of the *AMPK* pathway induced by betaine may represent a significant target for gastric cancer therapy.

## 4. Materials and Methods

### 4.1. Patients and Specimens

We collected plasma (with EDTA anticoagulant) from 334 GC and healthy people and utilized them to conduct untargeted metabolomics assays. Patient information is presented in Tables S1–S5.

Cancer tissues and corresponding paracancerous samples of six pairs of GC patients were collected according to the current diagnostic criteria. GC patients were admitted to the hospital for the first time without radiotherapy and had gastric adenocarcinoma (precancerous lesions, gastric cancers of different progressive stages) with a clear pathologic or endoscopic diagnosis. Tissues were collected from GC and paracancerous gastric mucosal tissues more than 5 cm away from the edge of the tumor, and the procedure of sample collection was approved by the Medical Ethics Committee of Zhongda Hospital, affiliated with Southeast University, and informed consent was signed with the patients.

### 4.2. Cell Proliferation Assays

Cell viability was assessed using the CCK8 (Cellcook, Guangzhou, China) assay. Cells were seeded in a 96-well plate at a density of 8 × 10^3^ cells per well and incubated in a CO_2_ incubator at 37 °C for 24 h; 10 μL of the Cell Counting Kit-8 mixture was added to each well, and the plate was further incubated for 1 h. The absorbance of the samples was then measured at a wavelength of 450 nm using a spectrophotometer.

### 4.3. Migration and Invasion Assays

For cell migration assays, cells were seeded into upper chambers (Corning, Corning, NY, USA). A total of 600 μL of complete medium (RPMI 1640, Gibco, CA, USA) with 20% FBS (VivaCell, Shanghai, China) was added to the lower chamber. Transmigrated cells were fixed in 4% paraformaldehyde (15 min) (Servicebio, Wuhan, China) after 24 h. Samples were examined under a microscope (Zeiss, Oberkochen, Germany), and a field of view was selected for cell counting. For the invasion assay, 50 μL of a Matrigel (BD Biosciences, Franklin Lake, NJ, USA) and serum-free medium mixture (1:8 ratio) was used in the upper chamber and polymerized at 37 °C for 30 min. The remaining steps are identical to those of the cell migration assays.

### 4.4. RNA Sequencing Analysis

In this experiment, six pairs of gastric cancer and paracancerous tissues were subjected to high-throughput sequencing using the double-end sequencing mode of illumina Novaseq6000 sequencing platform (Shanghai Jingneng, Shanghai, China). According to the distribution of low-quality scores at the end of illumina sequencing data, we used Skewer software (v0.2.2) to dynamically remove the junctions and low-quality fragments from the 3′ end of the sequencing data, and FastQC software (v0.11.9) to analyze the pre-processed data for quality control. Differential gene expression analysis between groups was conducted using DESeq2 R package (v1.34.0).

### 4.5. Single-Cell RNA Data Analysis

Utilizing data obtained from the GEO database (GSE184198), we conducted an analysis of the fluctuations. R software (v4.3.2) was used to perform the analysis. All scRNA-seq data were analyzed following data integration using the Seurat software package (v5). We performed data quality control using the criteria of 200 < nFeature_RNA < 2500 and percent.mt < 20. ElbowPlot was used to rank the principal components. t-SNE algorithm was used to nonlinearly reduce the dimensionality of the principal components.

### 4.6. Reverse Transcription-Quantitative (RT-q) PCR

Total RNA from cells was extracted using Trizol reagent (Epizyme, Shanghai, China) according to the manufacturer’s protocol; reverse transcription of RNA was performed using a PrimeScript RT kit (Vazyme, Nanjing, China), and qPCR quantification was performed using TB Green Premix Ex TaqII (Vazyme, Nanjing, China). Relative mRNA levels were determined by the 2^−ΔΔCT^ method, and β-actin was standardized as an internal control. Gene primers are listed in Table S5. The experiments were repeated three times independently.

### 4.7. Western Blot Analysis

The cells were isolated in ice-cold RIPA buffer (Epizyme, Shanghai, China). The proteins were separated using 10% SDS-PAGE and electroblotted onto a polyvinylidene fluoride (PVDF) membrane (Immobilon^®^-P) after quantitative determination of the protein concentration. The membranes were blocked in 5% non-fat milk for 1 h, then incubated overnight at 4 °C with primary antibodies anti-AMPKα (1:1000, Huaan, Hangzhou, China), anti-AMPK (1:5000, Huaan, Hangzhou, China), and anti-β-actin (1: 40,000, Huaan, Hangzhou, China). Subsequently, the secondary antibodies (anti-mouse/anti-rabbit) were applied, incubating membranes at room temperature for 2 h. Omni-ECL^TM^pico pight chemiluminescence kit (Epizyme, Shanghai, China) was used to analyze the protein brand under an enhanced chemiluminescence system (Tanon, Shanghai, China). Band intensities from three biological experiments were quantified by densitometry using ImageJ software (V1.8.0.112).

### 4.8. Metabolomics Sample Preparation

Plasma samples were thawed at room temperature. Two hundred microliters of cold methanol/acetonitrile (4 °C, *v*/*v* = 1:9, Merk, Darmstadt, Germany) were added to fifty microliters of plasma, followed by vortexing for 2 min, sonication for 1 min, and centrifugation at 12,000 rpm for 15 min at 4 °C. The supernatant was transferred to a sample vial for subsequent analysis.

### 4.9. Untargeted Metabolomics Analysis

Plasma metabolomics were analyzed using UPLC-Orbitrap-HRMS with a resolution of 240,000. Metabolite separation was performed using a C18 reversed-phase column (ACQUITY UPLC HSS T3 Column, 100 Å, 1.8 µm, 2.1 mm × 100 mm, 1/pk, Waters) with mobile phase A (0.1% formic acid aqueous solution in the positive ionization mode or 0.1% NH_3_-20 mM NH_4_Ac in the negative ionization mode) and mobile phase B (acetonitrile). The flow rate of the mobile phase was maintained at 0.3 mL/min, and the column temperature was kept at 40 °C. The injection volume was 5 μL. The positive and negative H-ESI were set at 3.4 kV and −2 kV, respectively. The normalized collision energy (NCE) was 30%. The gas pattern was static. Sheath gas was set at 40, auxiliary gas at 10, purge gas at 0, and ion transfer tube temperature at 350 °C. The gas flow rate of the ion transfer tubes was set at 0.5 L/min. Gasification temperature was set at 400 °C. Monitoring mode was set to data-dependent mode, with the number of dependent scans set to 10.

### 4.10. Data Processing and Analysis

The metabolomics data were processed online using Compound Discoverer 3.1, and metabolite annotations were performed using the mzCloud, mzVault, and ChemSpider databases. The vacant values were filled using the “Fill Gaps” function of the software, and the samples were normalized based on peak areas. Subsequently, SIMCA-P software (v14.1) was employed to analyze the normalized data. Characteristics with |Log2FC| > 1.0 and *p*-values < 0.05 across multiple groups were identified as differential metabolites.

### 4.11. Statistical Analysis

Statistical analyses were conducted using GraphPad Prism (v 6.02). Metabolomics data processing included unsupervised pattern recognition via principal component analysis (PCA) and metabolic pathway impact analysis (pathway topology > 0.1) using MetaboAnalyst 5.0. Inter-group comparisons used (i) two-tailed Student’s *t*-test for pairwise analysis; (ii) one-way ANOVA with Dunnett’s correction for multiple comparisons against healthy controls. Results are expressed as mean ± SD.

## 5. Conclusions

Metabolomics analyses revealed that the plasma concentrations of betaine, oleamide, pentadecanoic acid, and arachidonic acid were significantly decreased in patients with gastric cancer (GC). The combined analysis of transcriptomics, including single-cell transcriptome, with the metabolome indicated that pathways related to one-carbon metabolism (1CM), such as amino acid metabolism, are significantly altered in gastric cancer. Further biological experiments demonstrated that betaine, a crucial “one-carbon donor,” could inhibit the progression of GC by suppressing the activity, invasion, and migration of GC. And the addition of betaine can also significantly promote the expression of *AMPKα* and *AMPKβ* genes in two types of gastric cancer cells (HGC-27 and AGS) and activate the phosphorylation of *AMPKα*. This study provides new data to enhance our understanding of the pathogenesis of GC and to explore potential therapeutic approaches.

## Data Availability

The data that support the findings of this study are openly available in figshare at https://figshare.com/, DOI: 10.6084/m9.figshare.28771166.

## Supplementary Materials

The following supporting information can be downloaded at: https://www.mdpi.com/article/10.3390/ijms26083841/s1.

## Abbreviations

The following abbreviations are used in this manuscript:

GC	Gastric cancer
1CM	1-carbon metabolism
me-THF	5,10-Methylenetetrahydrofolate
PCA	principal component analysis
GEO	Gene Expression Omnibus
