# Supplementary material for: Identifying Aberrant 1CM-Related Pathways by Multi-Omics Analysis and Validating Tumor Inhibitory Effect of One-Carbon Donor Betaine in Gastric Cancer
Source: Int J Mol Sci. 2025 Apr 18;26(8):3841. doi: 10.3390/ijms26083841 (PMC12027648; doi:10.3390/ijms26083841)
Supplement: Supplementary file 1 [file ijms-26-03841-s001.zip › ijms-3546985-supplementary.pdf]

Identifying Aberrant 1CM-related Pathways by Multi-Omics Analysis and Validating Tumor Inhibitory Effect of One-carbon Donor Betaine in Gastric Cancer

Li Jie[1], Liu Huan[1], Yang Panpan[1], Zhu Feng[2], Shen Fei[2], Liang Geyu[1, *]

[1]Key Laboratory of Environmental Medicine Engineering, Ministry of Education, School of Public Health, Southeast University, Nanjing, 210009, Jiangsu, China; [2]Jiangsu Prov Ctr Dis Control & Prevent, 172 Jiangsu Rd, Nanjing 210009, Peoples R China.

Table S1 Clinical features of samples 2
Table S2 Statistical analysis of metabolites corrected for sex and age 2
Table S3 Stratifying differential metabolites by sex and age 3
Table S4 Clinical features of GC samples 3
Table S5 Primer sequences of mRNA 4
Fig.S1 Neg PCA for untargeted metabolomics 5
Fig.S2 Pos PCA for untargeted metabolomics 5
Fig.S3 The results of the nFeature_RNA values, nCount _RNA values, and cent.mt values. 6
Fig.S4 The PC1 to PC12 principal components and the genes that play a major role. 6
Fig.S5 Relative expression of metabolite betaine in gastric cancer progression. 6

Table S1 Clinical features of samples

Clinical feature		GC samples (N=164)	Health samples (N=170)	*P*
Gender				< 0.05
	Female	35	87	
	Male	129	83	
Age				< 0.05
	≤65	101	132	
	> 65	63	38	

Table S2 Statistical analysis of metabolites corrected for sex and age

Metabolites	*P*
**betaine**	**< 0.001**
**oleamide**	**< 0.001**
**Arachidonic acid**	**0.004**
**Pentadecanoic acid**	**< 0.001**
**DL-Carnitine**	**< 0.001**
**13-HOTrE**	**< 0.001**
**luteolin**	**< 0.001**
**Citric acid**	**< 0.001**
**Ethyl myristate**	**< 0.001**
**Docosahexaenoic Acid**	**< 0.001**
**D-(-)-Quinic acid**	**0.031**
**Ornithine**	**< 0.001**
**Spermine**	**< 0.001**
**Palmitoleic acid**	**< 0.001**
**Octadecenoic acid**	**< 0.001**
**D-Erythro-sphingosine 1-phosphate**	**< 0.001**
**Cortisol**	**< 0.001**
**Adenosine 5'-monophosphate**	**0.011**
**Pyruvate**	**< 0.001**
**Lactic acid**	**< 0.001**
**D-(+)-Malic acid**	**< 0.001**
**Fumaric acid**	**< 0.001**
**Glycyl-L-leucine**	**< 0.001**
**DL-Arginine**	**< 0.001**
**D-(+)-Proline**	**< 0.001**
**Palmitoylcarnitine**	**< 0.001**
**L-Pyroglutamic acid**	**< 0.001**
**L-Histidine**	**< 0.001**
**Decanoylcarnitine**	**< 0.001**
**DL-Glutamine**	**< 0.001**
**Spermidine**	**< 0.001**

Table S3 Stratifying differential metabolites by sex and age

Metabolites	*P*		*p*	
	Male(cancer/health)	Female(cancer/health)	≤65（cancer/health）	＞65（cancer/health）
betaine	< 0.001	< 0.001	< 0.001	< 0.001
oleamide	< 0.001	0.003	< 0.001	< 0.001
Arachidonic acid	< 0.001	< 0.001	< 0.001	< 0.001
Pentadecanoic acid	< 0.001	< 0.001	< 0.001	< 0.001
DL-Carnitine	< 0.001	< 0.001	< 0.001	< 0.001
13-HOTrE	< 0.001	< 0.001	< 0.001	< 0.001
luteolin	< 0.001	< 0.001	< 0.001	< 0.001
Citric acid	< 0.001	< 0.001	< 0.001	< 0.001
Ethyl myristate	< 0.001	< 0.001	< 0.001	< 0.001
Docosahexaenoic Acid	< 0.001	< 0.001	< 0.001	< 0.001
D-(-)-Quinic acid	< 0.001	< 0.001	< 0.001	< 0.001
Ornithine	< 0.001	< 0.001	< 0.001	< 0.001
Spermine	< 0.001	< 0.001	< 0.001	< 0.001
Palmitoleic acid	< 0.001	< 0.001	< 0.001	< 0.001
Octadecenoic acid	< 0.001	< 0.001	< 0.001	< 0.001
D-Erythro-sphingosine 1-phosphate	< 0.001	< 0.001	< 0.001	< 0.001
Cortisol	< 0.001	< 0.001	< 0.001	< 0.001
Adenosine 5'-monophosphate	< 0.001	< 0.001	< 0.001	< 0.001
Pyruvate	< 0.001	< 0.001	< 0.001	< 0.001
Lactic acid	< 0.001	< 0.001	< 0.001	< 0.001
D-(+)-Malic acid	< 0.001	< 0.001	< 0.001	< 0.001
Fumaric acid	< 0.001	< 0.001	< 0.001	< 0.001
Glycyl-L-leucine	< 0.001	0.007	< 0.001	< 0.001
DL-Arginine	< 0.001	< 0.001	< 0.001	0.001
D-(+)-Proline	< 0.001	0.001	< 0.001	0.008
Palmitoylcarnitine	< 0.001	< 0.001	< 0.001	< 0.001
L-Pyroglutamic acid	0.017	< 0.001	< 0.001	0.003
L-Histidine	< 0.001	< 0.001	< 0.001	< 0.001
Decanoylcarnitine	< 0.001	0.003	< 0.001	< 0.001
DL-Glutamine	< 0.001	< 0.001	< 0.001	< 0.001
Spermidine	< 0.001	0.003	< 0.001	< 0.001

Table S4 Clinical features of GC samples

Clinical feature	group	Case (N=164)
Gender	Female	35
	Male	129
Age	≤65	101
	＞65	63
TNM Stage	I	44
	II-III	109
	IV	36
Lauren	Intestinal type	70

	Diffuse type	53
	Mixed type	41
Tumor size	≤5cm	114
	＞5	50
Lymphatic node transfer	N0	64
	N1	32
	N2	25
	N3	39
	N_X	4
Tumor Grade	G1	22
	G2	70
	G3	72
Location	Cardia	38
	Fundus	23
	Body	52
	Antrum	51

Table S5 Primer sequences of mRNA

RNAs	Primer sequences (5′ to 3′)
AMPKα-F	TTGAAACCTGAAAATGTCCTGCT
AMPKα-R	GGTGAGCCACAACTTGTTCTT
AMPKβ-F	CCACTCCGAGGAAATCAAGGC
AMPKβ-R	CTGGGCGGGAGCTTTATCA
β-actin-F	CCTCG CCTTT GCCGA TCC
β-actin -R	GGATC TTCAT GAGGT AGTCA GTC

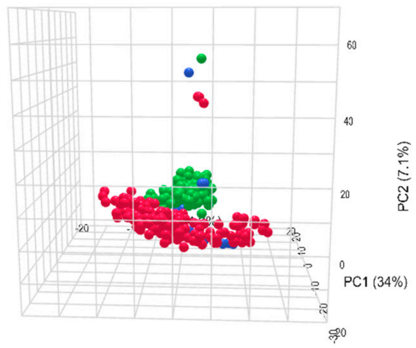

Fig.S1 Neg PCA for untargeted metabolomics

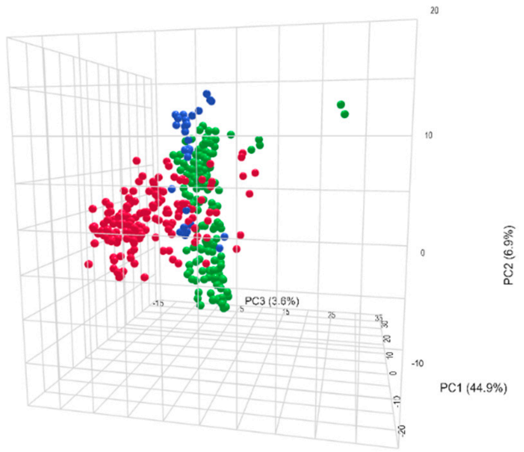

Fig.S2 Pos PCA for untargeted metabolomics

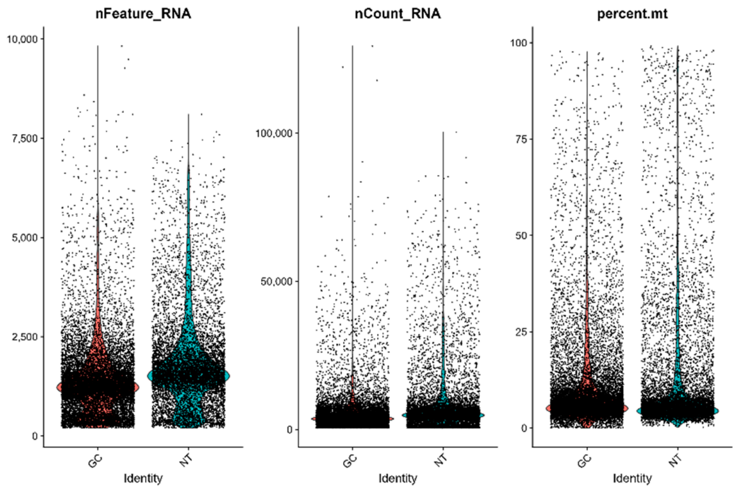

Fig.S3 The results of the nFeature_RNA values, nCount _RNA values, and cent.mt values.

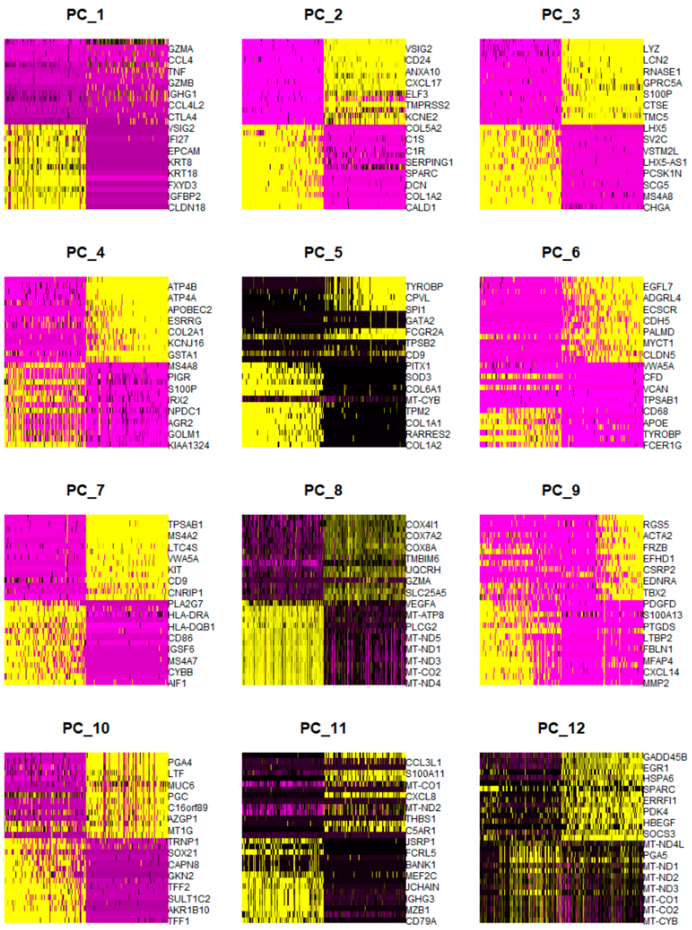

Fig.S4 The PC1 to PC12 principal components and the genes that play a major role.

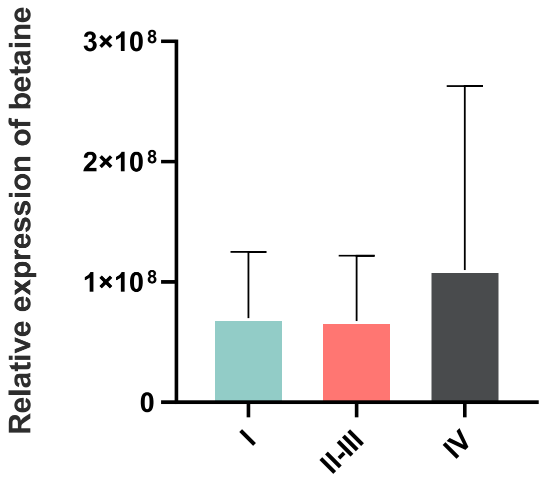

Fig.S5 Relative expression of metabolite betaine in gastric cancer progression.
